# The role of unidentified bright objects in the neurocognitive profile of neurofibromatosis type 1 children: a volumetric MRI analysis

**DOI:** 10.1007/s13760-023-02381-0

**Published:** 2023-09-21

**Authors:** Martina Di Stasi, Sirio Cocozza, Sara Buccino, Chiara Paolella, Linda Di Napoli, Alessandra D’Amico, Daniela Melis, Lorenzo Ugga, Gianmichele Villano, Manuel Ruocco, Iris Scala, Arturo Brunetti, Andrea Elefante

**Affiliations:** 1 Department of Advanced Biomedical Sciences, University of Naples “Federico II”, Via Pansini 5, 80131 Naples, Italy; 2 Department of Diagnostic and Interventional Neuroradiology, University Hospital “San Giovanni di Dio e Ruggi di Aragona”, Salerno, Italy; 3 Radiology Unit, “Tortorella” Private Clinic, Salerno, Italy; 4 Department of Medicine, Surgery and Dentistry, University of Salerno, Salerno, Italy; 5 Department of Maternal and Child Health, Federico II University Hospital, Naples, Italy

**Keywords:** Neurofibromatosis type1, MRI, UBOs, Cognitive impairment

## Abstract

**Purpose:**

Cognitive impairment is described in 80% of Neurofibromatosis type 1 (NF1) patients. Brain focal areas of T2w increased signal intensity on MRI, the so-called Unidentified Bright Objects (UBOs) have been hypothesized to be related to cognitive dysfunction, although conflicting results are available in literature. Here, we investigated the possible relation between UBOs’ volume, cognitive impairment, and language disability in NF1 patients.

**Material and methods:**

In this retrospective study, clinical and MRI data of 21 NF1 patients (M/F = 12/9; mean age 10.1 ± 4.5) were evaluated. Brain intellectual functioning and language abilities were assessed with specific scales, while the analyzed MRI sequences included axial 2D-T2-weighted and FLAIR sequences. These images were used independently for UBOs segmentation with a semiautomatic approach and obtained volumes were normalized for biparietal diameters to take into account for brain volume. Possible differences in terms of normalized UBOs volumes were probed between cognitively affected and preserved patients, as well as between subjects with or without language impairment.

**Results:**

Patients cognitively affected were not different in terms of UBOs volume compared to those preserved (*p* = 0.35 and *p* = 0.30, for T2-weighted and FLAIR images, respectively). Similarly, no differences were found between patients with and without language impairment (*p* = 0.47 and *p* = 0.40, for the two sequences).

**Conclusions:**

The relation between UBOs and cognition in children with NF1 has been already investigated in literature, although leading to conflicting results. Our study expands the current knowledge, showing a lack of correlation between UBOs volume and both cognitive impairment and language disability in NF1 patients.

## Introduction

Neurofibromatosis type 1 (NF1, OMIM #162200) is the most common neurocutaneous disorder, affecting 1/2700 live births worldwide [1], with a complete penetrance and without a known gender or ethnicity predilection [2]. It is caused by a germline heterozygous mutation in the *NF1* gene, encoding for the tumor-suppressor protein neurofibromin, with a pattern of autosomal dominant inheritance or de novo mutations in 42% of individuals [1].

This condition is characterized by a wide range of clinical manifestations, as highlighted in the recently published NIH Revised Diagnostic Criteria [3], with the most frequent being the presence of *café-au-lait* macules (CALMs), freckling in the axillary or inguinal region, neurofibromas of any type or plexiform neurofibromas, iris Lisch nodules, optic pathway glioma, and bone lesions (e.g. sphenoid dysplasia, bowing of the tibia, pseudarthrosis of long bones). With reference to Central Nervous System (CNS) involvement, cognitive dysfunction represents the most significant complication in NF1 children, with about 80% of patients showing moderate to severe impairment in at least one area of cognitive functioning [4]. Indeed, previous studies demonstrated that the mean IQ score, as measured by means of the Wechsler Intelligence Scales for Children-Revised (WISC-R), tends to reach lower ranges in NF1 patients, falling within one standard deviation of the general population [5, 6]. Furthermore, deficits in specific skills, including but not limited to visuospatial ability, executive function, expressive and receptive language, have also been reported in the literature [4, 7–9], with a relatively wide proportion of patients (from 38 to 50%) that meet diagnostic criteria for ADHD [10].

From a neuroimaging standpoint, the main brain parenchymal alteration in NF1 is the presence of focal areas of T2-weighted hyperintensity defined Unidentified Bright Objects (UBOs), also known as Focal Areas of Signal Intensity. The correlation between UBOs and a decrease in cognition and behavioral skills [11] has been extensively indagated, achieving conflicting results [12]. In particular, while some studies suggested a relation between the presence of UBOs in thalamus and striatum and impairment in calculation and behavioral performances, respectively [13], other failed to prove such correlation [14]. Similarly, with reference to intellectual performances, some studies showed that thalamic [15, 16] and cerebellar [17] UBOs were associated with lower IQ scores, although these correlations are not consistent between the different studies published [14]. Finally, it is noteworthy to mention that a possible association between the presence of OPG and worst cognitive functions in NF1 patients has been also suggested [18].

Very recent evidence seems to suggest a correlation between UBOs volume, calculated with a full automated technique, and reading abilities [19]. Given that cognitive dysfunctions, especially related to the language and social behavior domains, have a serious impact on NF1 patients’ quality of life [11], further data are absolutely required not only to further understand the causal pathophysiological mechanisms behind the development of these changes but also and especially in the identification of potential diagnostic biomarkers of cognitive involvement in NF1. Given this background, in the current study we tried to investigate the possible relation between UBOs’ volume, cognitive impairment and language disability in NF1 patients.

## Materials and methods

### Participants

This retrospective single-center study has been performed at the University of Naples “Federico II” in compliance with the Helsinki Declaration, with all patients (or legal guardians in case of subjects with less than 18 years) that provided a written consent to the execution of the imaging exams and collection of clinical data for research purposes.

**Fig. 1:**
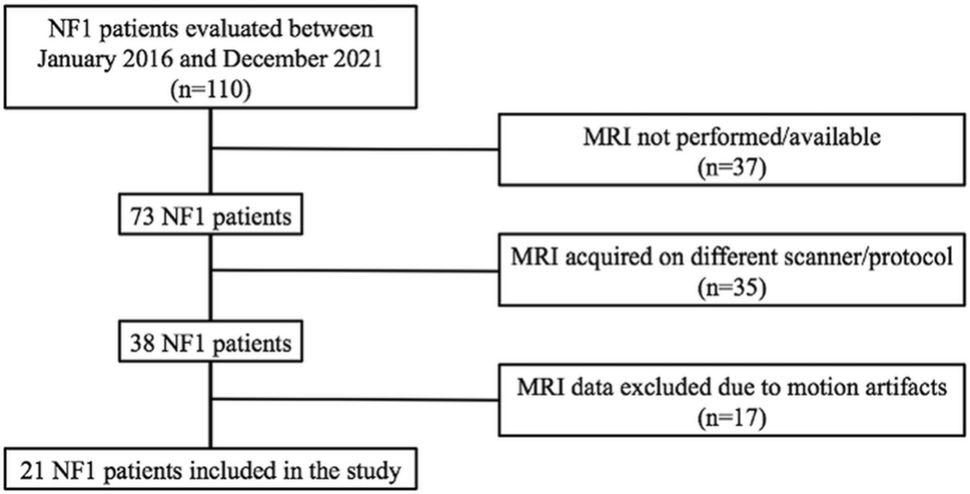
Flow diagram showing the patients’ selection procedure

A flow-diagram for the selection of the included subjects is available in Fig. 1. Briefly, inclusion criteria were the following: fulfillment of the revised diagnostic NF1 criteria [3], ability to undergo a neuropsychological examination, availability of brain MRI data acquired on the same scanner and with the same acquisition protocol. On the other hand, patients with significant artifacts on neuroradiological examination, concurrent neurologic disorders beyond the spectrum of NF1 as continuing seizures, serious psychiatric illness and previous neurosurgery or coexisting brain neoplasm (except optic pathway glioma—OPG—or small pilocytic astrocytoma) were excluded from the study.

### Clinical data

For all subjects, general clinical information were collected by a board-certified pediatric clinical geneticist (IS, with more than 10 years of expertise).

To assess general intellectual functioning and the presence of language deficit the following scales have been used: the Leiter R scale [20] the Wechsler Preschool and Primary Scale of Intelligence—Fourth Edition (WPPSI-IV) for children aged < 6 years, and the Wechsler Intelligence Scale for Children IV (WISC-IV) [21] for children aged ≥ 6 years. Physicians who administered the tests were blinded to the MRI findings.

Patients were stratified into two groups (affected/unaffected) according to the presence or absence, respectively, of cognitive impairment (defined as present for IQ scores < 70 or if the subject was unable to perform the test [22]), language deficit (defined present for clinical evidence of a delay in expressive language acquisition, a Verbal Comprehension Index sub-score of the WISC-IV scale < 85 or if the subject was unable to perform the test [23]) and OPG (absence/presence).

### MRI data acquisition

All MRI data were acquired on the same 1.5 T scanner (Gyroscan Intera, Philips Medical System, Best, Netherlands) with a standard 16-channel head coil. The acquisition protocol included, along with other clinically routine acquired sequences (e.g. diffusion-weighted imaging, sagittal and/or coronal T2-weighted sequences, MR-angiography sequences for the study of the intracranial vasculature system in clinical suspect of stenoses and Moya Moya syndrome, etc.) an axial Fluid Attenuated Inversion Recovery (FLAIR) sequence (TE = 100 ms; TR = 10805 ms; slice thickness = 4 mm; no gap) and an axial Turbo Spin Echo (TSE) T2-weighted sequence (TE = 98 ms; TR = 6500 ms; slice thickness 2 mm; no gap) for the evaluation of the UBOs, and a Short Tau Inversion Recovery (STIR) T2-weighted sequence (TE = 104 ms; TR = 9530 ms; slice thickness = 3 mm; no gap) for the identification of lesions of the optic-diencephalic region.

### MRI data analysis

All MRI data were evaluated in consensus by two readers (MDS and SC, board-certified neuroradiologist both with more than 6 years of expertise in neuroimaging).

**Fig. 2:**
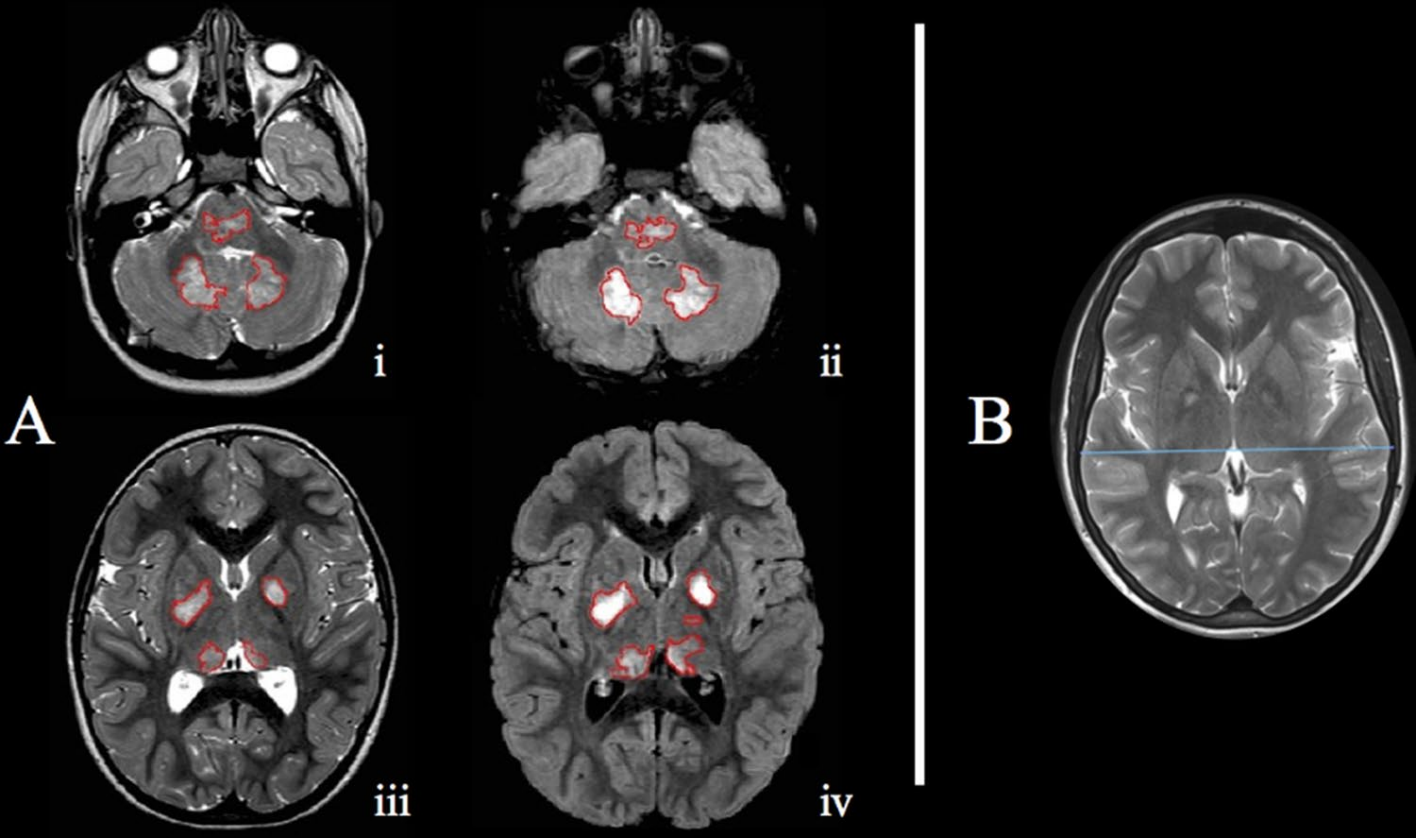
Image showing the MRI metrics obtained in this study. **A** infra- (i; ii) and supratentorial (iii; iv) UBOs segmentation masks on T2-weighted (left) and FLAIR (right) images of a 6 years-old patient. **B** an example of the biparietal diameter (blue line) used for normalization purposes, measured at the level of Monro foramina in a 16 years-old patient (colour figure online)

Both axial TSE T2-weighted and FLAIR images were used, independently, for the UBOs segmentation, with the additional aim of probing if significant differences between the two sequences were present in the identification of these lesions. The readers evaluated randomly FLAIR and T2-weighted images, and after a washout period of 30 days the other sequence was segmented. The segmentation procedure was carried out using a semiautomatic approach (Jim 8, Xinapse Systems, Northants, UK), and total UBOs’ volume, expressed in milliliters, was obtained for each subject (Fig. 2A). For normalization purposes, biparietal diameters on T2w images were also recorded to normalize for head size (Fig. 2B), and UBOs volumes were divided for this value.

The presence and extent of OPG were determined according to the modified Dodge classification (mDC) [24], which, briefly, proposes an MRI-based method to categorize tumors in greater detail also considering functional visual risk. The new classification introduces three subcategories of optic nerves involvement (unilateral, bilateral, and chiasmatic junction), two categories of chiasma site (central or asymmetric), three categories of optic tracts extension (symmetric or asymmetric and diffuse posterior tracts) and considers the neoplastic involvement of hypothalamic region and leptomeningeal spread.

### Statistical analysis

Possible differences in terms of UBOs volumes (normalized for biparietal diameters) between cognitively affected and preserved patients, as well as differences between subjects with or without language impairment were tested via General Linear Model analyses, corrected for age and sex. Similarly, given that previous data suggested that individuals with OPG had significantly more UBOs than individuals without OPG [25], we investigated if a similar feature was present in our group. Finally, possible differences in terms of volumes between measurement evaluated on FLAIR or T2-weighted images were tested via paired *t* test.

All analyses were performed using the Statistical Package for Social Science (SPSSv25.0, IBM corp.) with a significance level set for *α* = 0.05.

## Results

Following inclusion and exclusion criteria and a review of MRI data, a final number of 21 NF1 patients (M/F = 12/9; mean age 10.1 ± 4.5, range 5–18) referred to our Clinical Genetic Unit were included in this study. From a clinical perspective, with the complete list of findings that is available in Table 1, we found that 14 out of 21 subjects (66.7%) showed a cognitive deficit, while in 9 patients (42.9%) an impairment of the language domain was present.

**Table 1:** Clinical and demographic information of the enrolled patients

	NF1 (all patients, *n* = 21)	NF1 patients with cognitive impairment (*n* = 14)	NF1 patients with language deficit (*n* = 9)	NF1 patients with OPG (*n* = 11)
Age (mean ± SD)	10.1 ± 4.5	9.4 ± 4.1	10.4 ± 5.4	9.7 ± 4.2
Sex (M/F)	12/9	9/5	7/2	6/5
Macrocrania	7/21	7/14	4/9	2/11
Plexiform neurofibromas	3/21	1/14	1/9	2/11
Bone anomalies	3/21	2/14	2/9	1/11

Age is expressed in the year

*NF1* neurofibromatosis type 1, *SD* standard deviation

On MRI, all patients (21/21, 100.0%) proved to have at least one UBO. On the other hand, 11 patients out of 21 (52.4%) proved to have an OPG, with more than half of these subjects (6/11) that showed an mDC grade equal to Ia, while the remaining patients that scored either a grade 2b (4/11) or 4b (1/11).

A complete list of the volumetric analysis results is available in Table 2. When evaluating UBOs volumes, we found significantly higher lesional volumes when segmentation was obtained on FLAIR images compared to the T2-weighted images (8.4 ± 9.1 ml vs 7.2 ± 7.8 ml, *p* = 0.01). Nevertheless, when probing possible differences between cognitively affected and preserved patients, we failed to find significant differences between the two groups neither using FLAIR (normalized UBOs volume: 0.08 ± 0.07 vs 0.03 ± 0.02, *p* = 0.30) nor T2-weighted (0.07 ± 0.06 vs 0.03 ± 0.02, *p* = 0.35) sequences. Similarly, no differences emerged between patients with or without language impairment for the two sequences (0.08 ± 0.09 vs 0.05 ± 0.04, *p* = 0.40, and 0.07 ± 0.07 vs 0.04 ± 0.05, *p* = 0.47, for FLAIR and T2-weighted, respectively). Finally, no differences between patients with and without OPG emerged neither using FLAIR (0.08 ± 0.07 vs 0.04 ± 0.06, *p* = 0.20) nor T2-weighted (0.07 ± 0.06 vs 0.03 ± 0.05 ml, *p* = 0.16) sequences.

**Table 2:** Results of the volumetric analyses, divided by groups

	NF1 (all patients)		NF1 patients with cognitive impairment	NF1 patients without cognitive impairment		NF1 patients with language deficit	NF1 patients without language deficit		NF1 patients with OPG	NF1 patients without OPG	
UBOs volume (T2-weighted)	7.2 ± 7.8		9.1 ± 8.9	3.4 ± 3.1		9.5 ± 9.6	5.9 ± 6.2		9.6 ± 8.5	4.6 ± 6.5	
UBOs volume (FLAIR)	8.4 ± 9.1		10.8 ± 10.2	3.7 ± 3.2		11.6 ± 11.9	6.6 ± 5.7		10.9 ± 9.4	5.7 ± 8.4	
*p*-value		0.01			0.35^*^			0.47^*^			0.16^*^
					0.30^#^			0.40^#^			0.20^#^

Volumes are expressed in milliliters (mean ± standard deviation)

^*^Indicates the *p*-value for the T2-weighted comparison of normalized UBOs volume, while ^#^ indicates the *p*-value for the FLAIR comparison

## Discussion

Being present in approximately 70% of NF1 subjects, UBOs represent the most common intracranial finding in these patients, with the most frequent localizations being brainstem, globus pallidus, thalamus, internal capsule and cerebellum [26]. They tend to vary in number and size over time and in respect of different localization, showing in some cases a non-linear trend, with the number of affected brain regions being relatively high during childhood, followed by a trend of decrease during adolescence and an increase afterwards [27]. The exact nature of UBOs is not yet completely understood, mostly due to the relative paucity of histopathological data. An autoptic examination of the brain regions corresponding to T2-weighted hyperintensity seen at MRI examination in 3 NF1 patients showed spongiotic changes with fluid-filled vacuoles of 5–100 μm in the myelin sheath, without demyelination or axonal loss [28]. In the absence of large post-mortem datasets, several advanced MRI techniques have been used to investigate, from a neuroimaging perspective, macro- and microstructural alterations behind UBOs development and brain changes occurring beyond these features. Affected NF1 children are known to show an increased brain volume, often associated with macrocephaly, although no clear correlation with the cognitive changes have been found [29]. Furthermore, the volume of the corpus callosum (CC), the thalamus and the striatum seem to be increased in NF1 children, correlating with lower scores in academic achievement and visual-spatial and motor skills [30]. In addition, positive correlations were found between cognitive abilities, social skills, and the volume of subcortical structures (i.e. hippocampus, thalami, striatum, amygdala and accumbens nucleus) [31], although these results have not been replicated in a different, more recent, article [14]. Brain involvement is, clearly, not related only to gray matter (GM) in NF1, extending also to the white matter (WM) compartment. Indeed, it has been shown that NF1 patients undergo widespread microstructural WM changes, either in terms of increased apparent diffusivity coefficient (ADC) and decreased fractional anisotropy (FA) values, along with alterations in axial (AD) and radial diffusivity (RD), indicative of looser fiber packaging rather than demyelination [32]. Interestingly, these alterations seem to occur independently from the presence of the pathognomonic parenchymal UBOs. With reference to these latter features, one study aimed to characterize their nature by combining results from advanced white matter imaging such as Multi-Exponential T2 relaxation (MET2) and Neurite Orientation Dispersion and Density Imaging (NODDI), showing the presence of intracellular water molecules-pool with extracellular-like properties, endorsing the hypothesis of intramyelinic vacuolization [33].

Independently from their pathophysiology, the relation between UBOs and cognition in NF1 patients has been extensively indagated in literature, leading unfortunately to somehow conflicting results. In particular, while some studies showed no statistical differences in IQ scores or learning disabilities in patients with and without UBOs [34–36], other found that although the presence, number, and locations of UBOs seem not to influence the general cognitive status, thalamic and striatal localization seems to, respectively, impact calculation and behavioral performances [13]. In contrast with this result, a more recent study showed that thalamic UBOs seemed to not have a significant impact on cognitive functioning, in the absence of correlations between thalamic (or other subcortical structure volumes) and specific cognitive scores [14]. With specific reference to intellectual performances, different papers reported that UBOs can affect this clinical feature [15] [16] [17], while one study showed that the number of UBOs might predict sibling-referenced lowering IQ [37]. Other Authors have reported a relation between basal ganglia UBOs volume and brain volume ratio and siblings-pairwise Judgement Line Orientation deficit [38]. While thalamic lesions were associated with lower intellectual function in two separate studies [15] [16], cerebellar UBOs have been associated with worse scores on verbal IQ, full-scale IQ and visuospatial tests [17]. Nevertheless, these findings have been recently rediscussed, given that have not been consistently replicated [14].

In this study, we analyzed UBOs using two different conventional imaging techniques (T2-weighted and FLAIR sequences) by means of the semiautomatic segmentation method, to further investigate the possible relation between UBOs’ volume, cognitive impairment and language disability in NF1 patients. Our first result is that, compared to the T2-weighted sequence, FLAIR sequence can detect a higher and probably more reliable lesion volume. This result is not unexpected, given that FLAIR sequences are known to have higher sensitivity in the detection of myelin alterations, especially in lesions close to cerebrospinal fluid and adjacent to the GM [39, 40]. In line with these considerations, in a condition different from a pathophysiological standpoint but characterized by the presence of white matter lesions such as Multiple Sclerosis, lesion volume are known to be higher when FLAIR sequences are evaluated compared to T2w images [39].

When possible differences between affected and unaffected patients were probed in terms of UBOs volumes, we failed to find any significant differences, independently from how patients were stratified and which MRI feature was evaluated. Nevertheless, the lack of differences is not unexpected given the available literature. In particular, it is possible to hypothesize that the occurrence of cognitive impairment and language deficit might be more related to the widespread loss of normal-appearing WM microstructural integrity and abnormal neuronal connectivity, as already reported in NF1 patients [32]. This speculation is also supported by findings in another neuro-phacomatosis characterized by similar decreased neurological outcomes in intellectual skills and learning abilities, namely the Tuberous Sclerosis Complex (TSC), in which a similar pattern of conventional brain changes has been reported [41]. In particular, analogously to UBOs in NF1, neither tubers load nor their localizations seem to show a strong correlation with cognitive outcome in TSC patients, and it has been suggested that TSC symptoms may be contingent on abnormal connections independent from local alterations evident at conventional imaging [22]. This possible explanation is also supported by the findings obtained in a study that using NODDI (which reflects WM microstructure [42]) showed in TSC patients altered scores compared with controls even in normal-appearing brain tissue, with a slight correlation with the degree of mental retardation [43]. Similarly, cognitive deficits (i.e. lower IQ values evaluated with WISC IV) related to microstructural WM changes have been reported in conditions as congenital hypothyroidism [44]. These speculations are also applicable to language and other academic achievements, in which a more pronounced involvement of frontal lobes’ WM integrity in the setting of diffuse alterations has been reported as a possible determinant of the development of specific neurocognitive profile in NF1 patients [32]. Finally, our results are in line with a very recent study failing to observe a correlation between intellectual functioning, language deficit and UBOs volumes segmented with a fully automated method [19].

Although all these considerations are plausible from a pathophysiological standpoint, we cannot exclude that the lack of difference here observed might be related to the low numerosity of our sample, which is the main limitation of this study. Indeed, as often happens when dealing with rare disorders, only 21 NF1 patients have been included in this study, obviously limiting our statistical power in possibly observing a small difference between the different groups (as proven by a posthoc power calculation showing that 43 NF-1 patients would have been needed to reach a power of 80%). Nevertheless, it has to be stressed that all the subjects here included underwent the same standardized MRI protocol and clinical evaluation, to minimize possible differences in terms of acquisition parameters that might influence semiautomatic measurements as the ones here produced. Nevertheless, future prospective studies, conducted using a larger sample size also to compare more patients of the same age (given the changes of UBOs volumes over time) are warranted, to further validate the hypothesis, here corroborated, of an absence of a significant effect of UBOs in the development of cognitive impairment and learning disability in NF1 patients.

## Data Availability

Data will be made available upon reasonable request to the corresponding author.
